# Translation and validation into Spanish of the oral health self-efficacy scale in Chilean older adults

**DOI:** 10.1186/s12903-024-03889-4

**Published:** 2024-01-20

**Authors:** Fernanda Muñoz-Sepúlveda, Claudia Acevedo, Rodrigo Mariño, Cristina Sanzana-Luengo, Pablo Navarro, Víctor Beltrán

**Affiliations:** 1 Master Program in Dental Science, Dental School, Universidad de La Frontera, Temuco, 4811230 Chile; 2 Clinical Investigation and Dental Innovation Center (CIDIC), Dental School and Center for Translational Medicine (CEMT-BIOREN), Universidad de La Frontera, Temuco, 4811230 Chile; 3 Interuniversity Center for Healthy Aging (CIES), Santiago, Chile; 4 Melbourne Dental School, Faculty of Medicine, Dentistry and Health Sciences, University of Melbourne, Melbourne, VIC 3010 Australia; 5 Center for Research in Epidemiology, Economics and Oral Public Health (CIEESPO), Dental School, Universidad de La Frontera, Temuco, 4811230 Chile; 6 Postgraduate Program in Oral Rehabilitation, Dental School, Universidad de La Frontera, Temuco, 4811230 Chile; 7 Research Center for Dental Sciences (CICO), Dental School, Universidad de La Frontera, Temuco, 4811230 Chile; 8 Facultad de Ciencias de la Salud, Universidad Autónoma de Chile, Temuco, 4811230 Chile

**Keywords:** Oral health, Self-efficacy, Psychometric validation, Patient-reported outcomes, Ageing

## Abstract

**Background:**

Population ageing poses a challenge to improving the well-being of older adults, particularly in terms of oral health. Promoting self-efficacy in oral health behaviours is crucial for maintaining this population’s health and quality of life. The Oral Health Self-Efficacy Scale (OHSES) has been widely used to assess dental self-efficacy and is considered comprehensive and reliable. However, there is a need to validate OHSES for Spanish-speaking older adults. This study aimed to assess the reliability and validity of the translated questionnaire for use in the older Chilean population.

**Methods:**

A sample of 188 older adults, aged 60 years and above residing independently in the community, were recruited by accessing databases from the National Senior Citizen Service and various community organisations within the region of La Araucanía. The participants underwent comprehensive oral examinations and oral health interviews, focusing on variables such as OHSES, Oral health-related quality of life (OHIP-14Sp), assessment of remaining teeth, knowledge and attitudes toward oral health, and sociodemographic characteristics. The validity of the translated questionnaire was assessed through translation and cross-cultural adaptation, cognitive debriefing, and face and content validation. The psychometric properties of the questionnaire were evaluated through measures of internal consistency (Cronbach’s alpha), content validity (Content validity index), construct validity (factor analysis and Pearson correlation analysis), and test-retest reliability (intraclass correlation).

**Results:**

The Spanish version of OHSES demonstrated adequate face and content validity. The confirmatory factor analysis confirmed a two-factor scale with 7 items for a better fit. The scale demonstrated high internal consistency (Cronbach’s alpha = 0.821) and acceptable test–retest reliability (ICC = 0.411). Correlations were found between the OHSES score, the number of remaining teeth, knowledge and attitudes towards oral health, and the OHIP-14Sp (*p* < 0.01).

**Conclusions:**

This study confirms the validity of the Spanish version of the Oral Health Self-Efficacy Scale for older adults in Chile. The scale is expected to be helpful in assessing self-efficacy in dental interventions and collecting data for international comparisons. This research opens new dimensions in patient-reported assessment of oral health.

## Background

The global trend of population ageing presents a complex challenge: the enhancement of the quality of life of older adults. As people live longer, the focus on ensuring their well-being, health, social connections, and access to resources becomes fundamental for healthy ageing [1]. Maintaining good oral health is central to achieving this goal [2]. Moreover, including a patient’s psychological and social experiences concerning oral health could be an outcome of successful ageing [3, 4].

Promoting self-efficacy, is key to maintaining health and well-being, particularly for this age group, because it stimulates their physical abilities and the feeling of being able to actively participate in the search for solutions to their daily problems [5]. In the context of oral health, self-efficacy refers to an individual’s perceived confidence in their ability to organise and routinely execute the courses of action necessary to maintain the oral tissues in good condition [6]. Prominent levels of self-efficacy have positive implications for overall functioning and well-being [7]. It has been described that this psychological mechanism plays a pivotal role in influencing changes in oral health behaviours over time, serving as a link between knowledge and actions [8, 9]. Additionally, oral health self-efficacy has been linked to psychological well-being, predicting oral health related quality of life [10–12]. Self-efficacy has been noted to directly impact the ability to enact positive life changes and sustain the adoption of healthy behaviours that support successful ageing [13, 14]. Health promotion strategies often aim to enhance self-efficacy [15]. Oral health self-efficacy is an essential psychological factor that influences an individual’s ability to perform oral health behaviours effectively [13, 16–18]. Individual and population-based lifelong interventions are needed to maintain good oral hygiene practices; making healthy food choices; regular dental check-ups; and adherence to treatment [19]. Focusing solely on self-efficacy for specific aspects, like brushing, can overlook the broader role of self-efficacy in preventing tooth loss and maintaining overall oral health. Furthermore, integrating oral health self-efficacy, in clinical practice can enhance communication between patients and dentists, predict patient engagement, and enable shared decision-making and personalised treatment planning [13].

Several instruments have been created to measure oral health-related self-efficacy [17, 20–22]. This includes the Dental Self-Efficacy Scale (DSES) [20], the Self-efficacy scale for self-care (SESS) for periodontal disease patients [21], the scale of Oral Hygiene-related Self-Efficacy (OHSE) [17], more recently, the Geriatric Self-Efficacy Scale for Oral Health (GSEOH) [22] and the Oral Health Self-Efficacy Scale (OHSES) [23]. However, only the Oral Hygiene-related Self-Efficacy is translated to Spanish, to the best of our knowledge [24].

The OHSES was developed by Wiedenfeld and Kiyak and used in the Clinical Trials to Enhance Elders Oral Health (TEETH) aimed at reducing dental mortality in an older population with previous oral disease and a poor history of regular dental care [25]. This 8-item scale covers a wide range of factors that may influence older adults’ confidence in their ability to maintain good oral health [8]. OHSES has been widely employed in studies investigating behaviours related to preventing oral health diseases [8, 18].

This study chose the OHSES due to its proven reliability [8], purpose, content, and concise item count, facilitating its application both in epidemiological studies and during oral healthcare visit. However, before implementing the Spanish version of the OHSES, validation is necessary to ensure its readiness for practical use. This study aimed to assess the validity and reliability of the Spanish version of the OHSES (Sp) for use in Chilean older adults.

Over the last few decades, Chile is witnessing an epidemiological transition in oral health [26]. From a period marked by prevalent tooth decay and tooth loss, there has been a gradual shift toward better oral health outcomes. Several factors have contributed to this. However, challenges persist, additional efforts are needed to continue the focus on promoting oral health, equitable access to dental services, and preventive interventions to sustain and further advance this positive trend in Chile’s oral health landscape. In 2007, the Chilean ministry of health introduced the explicit health guarantees (Garantías Explícitas en Salud) in oral health to promote the prevention, care, and treatment of oral diseases, focusing on improving the oral health of the general population, especially, those groups that are vulnerable or have less access to health services [27]. Against this background, a validated OHSES(Sp) takes a significant role to empower and facilitate older Chileans to behavioural change to adopt healthier oral habits.

## Methods

This study was approved by the Research Ethics Committee of the Universidad de La Frontera, Temuco, Chile (Folio Number 082/22) and all subjects agreed to participate in the present study by signing an informed consent.

### Participants and setting

Chile is undergoing an advanced demographic transition, and within its regions, La Araucanía stands out with a substantial older adult population (18.9%) and distinct characteristics, including high vulnerability, a predominantly rural landscape, health inequities tied to social determinants, and a low frequency of preventive oral health check-ups [28–30]. La Araucanía encompasses 32 municipalities, each characterized by diverse demographics, with predominantly urban (35.1%), mixed (28.8%), and predominantly rural (36.1%) compositions [31].

As part of a broader study, older adults in La Araucanía received oral healthcare through a teledentistry protocol proposed by this article’s research team [32, 33]. Recruitment and subject selection were conducted via telephone by a collaborator, utilizing the Geriatric Dental Specialties Tele platform (TEGO) to collect and manage data and instruments [34]. Following Beltran et al., 2022 [34], inclusion criteria targeted older adults (60 and above) in need of primary dental care. Requirements for remote dental care included active pharmacological treatment for chronic diseases, the ability to comprehend verbal instructions, and sufficient mobility to board the mobile dental clinic. For safety reasons in a remote setting, individuals undergoing anticoagulant therapy, dialysis, and cancer treatment without medical authorization were excluded. Participants were recruited through databases of the regional branch of the National Senior Citizen Service (SENAMA) and community organizations, inviting those meeting the criteria and able to attend service centres in Freire and Temuco municipalities.

### Data collection

Data were collected using a cross-sectional study design. After an individual agreed to participate, the enrolment process consisted of a 20-minute telephone call. Data collected included:

- Sociodemographic data: The process of participant registration involved the collection of pertinent sociodemographic information, including details such as sex (male/female), age categorized into specific group (60–64, 65–74 and 75 years and older), ancestry (belonging to an Indigenous community), and educational attainment. The latter was classified by the number of years of study, with categories ranging from no scholar education, to 8 or fewer years, 9 to 12 years, and 13 or more years of education. Place of residence, classified according to national categorisation in predominantly urban, mixed, or predominantly rural [31].
- Oral health data: The OHIP-14 SP quality of life questionnaire [35], the Oral Health Self-Efficacy Scale, and knowledge and attitude towards oral health [8] were applied.

Oral Health Self-efficacy Scale: The original English version of the OHSES has 8 items under the subordinate categories of oral and general health self-efficacy, with six and two questions respectively [25]. All items are rated along a five-point Likert scale from “Not at all confident”, “Somewhat not confident”, “Neither confident nor unconfident”, “Confident”, and “Strongly confident” categories. The scale demonstrated high internal consistency (Cronbach’s alpha = 0.92) [23] and has been shown to have high test–retest reliability (Correlation coefficient = 0.88) [36].

Oral Health Impact Profile: The OHIP-14 has been widely used to measure adverse impacts of oral health on well-being. The OHIP-14 SP has been validated for measuring oral health-related quality of life in older Chilean Spanish-speaking populations [35]. The OHIP-14 SP consists of 14 items that cover seven domains of oral health: functional limitation, physical pain, psychological discomfort, physical disability, psychological disability, social disability, and handicap. The responses are given on a five-point Likert scale, ranging from “Never” to “Very often”.

Knowledge and attitude toward oral health: The knowledge and attitudes toward oral health were assessed through 43 questions used in previous studies [8, 18, 36]. The focus was identifying signs and symptoms of dental caries, periodontal disease, and oral cancer, and preventive measures for each condition. The responses for signs and symptoms were quantified using a binary “Yes” or “No” scale, while preventative measures were ranked on a 5-point scale ranging from “Very important” to “Not at all important”.

Attitudes were also measured via a 5-point scale, which evaluated seven items associated with the inevitability of oral disease in older people, the value of keeping natural teeth, and the effectiveness of preventive behaviours.

An overall score was calculated by summing up the correct responses of the knowledge items and the positive responses across all the attitude items. In contrast, higher scores reflected more knowledge and positive attitudes toward oral health.

c) Clinical data: After enrolment, an oral health examination was conducted by a trained and calibrated general dental practitioner in a mobile dental clinic, where a complete medical-dental-geriatric assessment was performed. Clinical data used in this analysis include the number of natural teeth. The data were collected between November 2022 and April 2023.

### Validation process

#### Translation and cross-cultural adaptation

This stage followed established guidelines [37–39] and began with two independent bilingual investigators translating the English questionnaire into Spanish (forward translation). Any language-related issues were addressed through discussion, leading to a consensus Spanish version (reconciliation). Subsequently, a native English translator, blinded to the concepts to be evaluated and to the original questionnaire document, performed an independent back-translation. Finally, a committee of six experts, comprising experienced researchers with doctorates (C.A, R.M and V.B) and the translators, reviewed all translations and created the pre-final version of the questionnaire for field testing (harmonization) [37, 38].

#### Cognitive debriefing

The field test of the pre-final version of the questionnaire was conducted in a convenience sample with at least 8 participants [37], obtained from SENAMA and older adults social clubs, targeting adults of 60 or more years from diverse backgrounds (Sex, age, educational level, place of residence). The participants were contacted by telephone and completed the pilot Oral Health Self-efficacy Scale. The comprehensiveness of the instrument was assessed by asking the respondent, immediately after answering each item, what they understood the question to mean (rephrase) and seeking for difficulties in understanding items (clarity, intelligible words, ease of response) [37]. This method enables the expert panel to identify concepts or constructs unique to English and make necessary corrections to prevent concept bias [39].

#### Face and content validation

An expert panel for the content and face validation was formed by 5 academics from Chilean state universities with a mean of 22.7 years of academic experience, all with postgraduate training in Dentistry, Public Health, Medical Sciences, Education and Communications. The panel had research expertise and was familiar with the construct that the questionnaire is designed to measure [39]. An online content validation form was sent to the experts with clear instructions. The Content Validity index was applied, specifically, the Item content validity index (I-CVI), measuring the appropriateness of scale items to accurately represent the intended construct. Experts assigned ratings to each questionnaire item using a scale where 1 signifies “not relevant”, 2 indicates “somewhat relevant”, 3 represents “quite relevant”, and 4 corresponds to “highly relevant and considered” [40]. The I-CVI score, calculated from expert ratings, considered the number of items rated as 3 or 4 divided by the total number of experts, with a score of 1 deemed appropriate [40].

#### Psychometric properties

Internal consistency was examined for the two 4-item OHSES subscales, using Cronbach’s alpha.

##### Test-retest reliability

A minimum sample of 32 participants [41, 42] was needed to measure the correlation of the OHSES recorded at a 10-day interval. The intraclass correlation coefficient (ICC) was used to obtain test–retest reliability.

##### Construct validity

The factor structure of the OHSES was assessed through factor analysis. If construct validity exists, the number of factors should approximate the number of dimensions assessed by the set of measures (i.e., two). For sample size calculation, we followed established guidelines [43, 44], including the criteria of total sample size (i.e. 100 subjects are deemed sufficient with a clear structure, but more is better), the cases to variables ratio (i.e. 10 subjects per variable), the ratio of cases to the number of factors (i.e. 20 subjects per factor), and the agreement between the sample and population solutions (i.e. a fixed variables-to-factors ratio of 8/2, with a high level of communality, and *k* values at least as high as 0.98). Based on these considerations, a minimum sample size of 150 participants was calculated to ensure an excellent recovery of the population factor solution [44].

Construct validity was also assessed by establishing convergent and divergent validity. For example, it was hypothesised that the number of remaining teeth and the knowledge and attitudes towards oral health would positively correlate with scores from the Spanish version of the Oral Health Self-efficacy Scale [8, 18, 36] (convergent validity) and the scores taken from the Spanish version of the Oral Health Impact Profile-14 (OHIP-14 Sp) would negatively correlate with the scale (divergent validity) [10].

### Statistical methods

Descriptive statistics in the form of frequency and percentage distributions were used to describe the background characteristics of the respondents. Reliability was calculated using Cronbach’s alpha and intraclass correlation coefficient.

From the 8 items of the OHSES, an exploratory factor analysis (EFA) was conducted using oblique rotation (oblimin) since it was assumed that the two factors were correlated. The sampling adequacy of the data for EFA was assessed using the Kaiser-Meyer-Olkin (KMO) statistic and Bartlett’s test of sphericity. To justify factor analysis, KMO values should exceed 0.50 and Bartlett’s test of sphericity should be significant [45]. A principal component analysis was run to extract the factors included in the questionnaire, and the criterion applied for selecting the optimal number of components was the Kaiser criterion (eigenvalue greater than one) [45]. A confirmatory factor analysis (CFA) using maximum likelihood (ML) estimation was conducted using the extracted factors. An acceptable model fit was defined by the following criteria: RMSEA (≤ 0.06), comparative fit index (CFI ≥ 0.95), and Tucker-Lewis index (TLI ≥ 0.95) [45, 46].

The Pearson correlation coefficient was computed to assess the relationship between the number of remaining teeth, Knowledge and attitudes toward oral health, OHIP-14 Sp, and OHSES. All analyses were conducted using SPSS version 23 and SPSS AMOS version 26. A p-value < 0.05 was considered significant.

## Results

The comparison of the two independent translations into Spanish analysed by the research team showed no conceptual or content differences. The cognitive debriefing was conducted in eleven older adults with a mean of 72.64 (SD 7.7) years and the comprehension of the questions ranged from 81.8 to 100% (Table 1). Items number 2 and 4 had the poorest comprehension scores. Given that the test was applied by telephone no incomplete questions were obtained, and few modifications of comprehensibility were added.

**Table 1:** Responses in the cognitive debriefing process

Patient Characteristics	Question understanding							
	Q1	Q2	Q3	Q4	Q5	Q6	Q7	Q8
**P1** Age 79 Educational Level: 9–12 years Place of residence: Rural	Yes	Yes	Yes	Yes	Yes	Yes	Yes	Yes
**P2** Age 64 Educational Level: 13 or more years Place of residence: Urban	Yes	Yes	Yes	Yes	Yes	Yes	Yes	Yes
**P3** Age 66 Educational Level: 13 or more years Place of residence: Urban	Yes	No	Yes	Yes	No	Yes	Yes	Yes
**P4** Age 85 Educational: Level 9–12 years Place of residence: Rural	Yes	Yes	Yes	Yes	Yes	Yes	Yes	Yes
**P5** Age 71 Educational Level: No scholar education Place of residence: Rural	Yes	Yes	Yes	No	Yes	Yes	Yes	Yes
**P6** Age 63 Educational Level: 13 or more years Place of residence: Urban	Yes	Yes	Yes	Yes	Yes	Yes	Yes	Yes
**P7** Age: 70 Educational Level: 8 or fewer years Place of residence: Urban	Yes	Yes	Yes	Yes	Yes	Yes	Yes	No
**P8** Age: 74 Educational Level: 9–12 years Place of residence: Rural	Yes	Yes	Yes	No	Yes	Yes	Yes	Yes
**P9** Age:85 Educational Level: 8 or fewer years Place of residence: Urban	Yes	No	Yes	Yes	Yes	Yes	Yes	Yes
**P10** Age: 74 Educational Level: 8 or fewer years Place of residence: Rural	Yes	Yes	Yes	Yes	Yes	Yes	Yes	Yes
**P11** Age 68 Educational Level: No scholar education Place of residence: Urban	Yes	Yes	Yes	Yes	Yes	Yes	Yes	Yes
Understanding (%)	100%	81.8%	100%	81.8%	90.9%	100%	100%	90.9%

There was 88.57% agreement for the face validity, with minor writing suggestions. The content validity index was almost satisfactory (I-CVI 0.98) (Table 2), with only one item rated as irrelevant by one expert. The expert indicated that the additional comment in question number 4 (do more than take a painkiller) could influence self-medication, so the statement was eliminated.

**Table 2:** Content validity index of the Oral health self-efficacy scale

	Expert 1	Expert 2	Expert 3	Expert 4	Expert 5	Experts in Agreement	Item CVI
Question 1: How confident are you about choosing what foods to avoid eating to prevent tooth loss?	4	3	4	4	4	5	1
Question 2: How confident are you about your ability to prevent tooth loss?	4	4	4	4	4	5	1
Question 3: How confident are you about your ability to take action if a filling cracks or falls out?	4	4	4	4	4	5	1
Question 4: How confident are you about your ability to take action if you have a toothache? (Do anything else than just taking a painkiller)	4	4	2	4	4	4	0.83
Question 5: How confident are you about your ability to floss correctly?	4	4	4	4	4	5	1
Question 6: How confident are you about Your ability to brush all surfaces of your teeth thoroughly?	4	4	4	4	4	5	1
Question 7: How confident are you about your ability to ask your doctor if you have questions or concerns about your medication?	4	4	4	4	4	5	1
Question 8: How confident are you about your ability to identify potentially dangerous side effects of the medication you take?	4	4	4	3	4	5	1
Proportion relevant	1	1	0.875	1	1	**Average** **I-CVI**	0.98

*I-CVI: Item content validity index

**Fig. 1:**
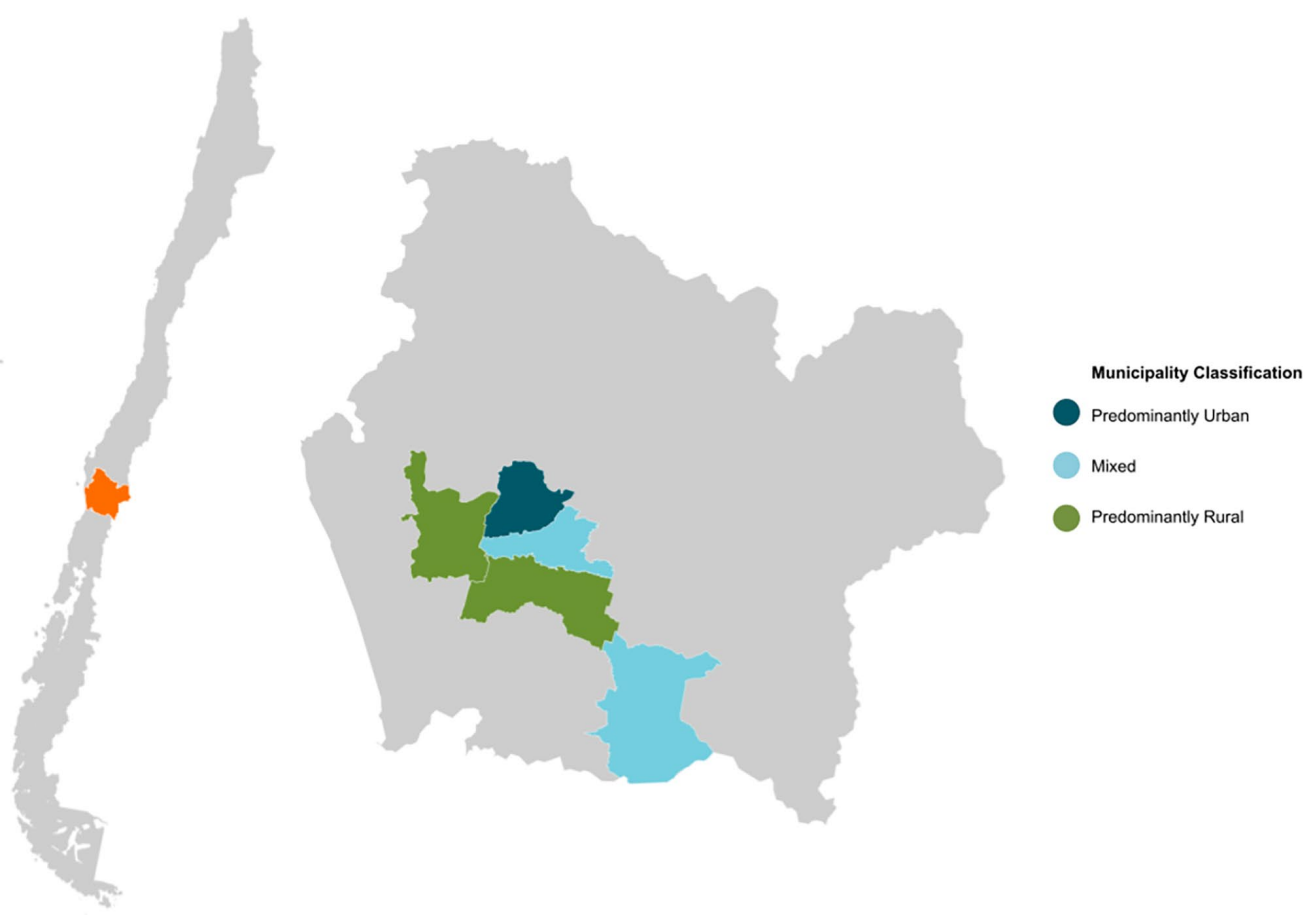
Map of Chile illustrating the selected municipalities in the La Araucanía region and their respective classifications

Data had been collected from 188 participants. The participants mean age was 71.41 (SD 7.21) years, and 52% of the sample was female. The respondent´s residences were 48.4% predominantly rural, 10.1% mixed, and 41.5% predominantly urban [31] (Fig. 1).

The mean Oral Health Self-efficacy score was 4.85 points [95% CI: 4.47–5.22]. For knowledge and attitudes toward oral health the mean was 25.82 points [95%CI: 24.72–26.93], with the knowledge mean being 22.31 [95%CI: 21.22–23.40] and the attitudes mean being 3.51 [95%CI: 3.29–3.73]. The OHIP-14 Sp assessment was 11.18 [95%CI: 10.01–12.35] points; and the remaining teeth were 14.73 [95% CI: 13.37–16.09]. Table 3 presents the sociodemographic and psychosocial characteristics of the sample.

**Table 3:** Participant socio-demographic characteristics, OHSES, OHIP-14 Sp, remaining teeth, knowledge and attitudes towards oral health

	Variables	n= 188	OHSES Mean (SD)	OHIP-14 Sp Mean (SD)	Remaining teeth Mean (SD)	Knowledge and attitudes Mean (SD)
**Sex**	Female	99	5.2 (2.26)	11.39 (7.73)	13.87 (9.26)	24.82 (7.28)
	Male	89	4.47(2.74)	10.57 (8.12)	15.42 (9.33)	25.54 (7.71)
**Mapuche ancestry**	Yes	85	4.27 (2.56)	12.99 (8.05)	10.31 (7.6)	23.76 (7.07)
	No	103	5.34 (2.39)	9.36 (7.43)	18.25 (9.05)	26.31 (7.64)
**Age groups**	60–64 years	38	5.23 (2.22)	12.08 (8.97)	16.67 (8.67)	25.21 (6.7)
	65–74 years	82	4.85 (2.47)	10.89 (7.9)	13.97 (9.0)	25.63 (7.86)
	75 years and more	67	4.69 (2.73)	10.38 (7.27)	14.36 (9.86)	24.54 (7.52)
**Education level**	No scholar education	16	5.44 (2.34)	13.31 (9.33)	14.12 (9.8)	25.75 (5.65)
	8 or fewer years of education	70	3.83 (2.6)	13.32 (8.43)	9.46 (7.62)	22.63 (8.2)
	9 to 12 years	59	4.9 (2.39)	9.98 (7.76)	15.69 (8.7)	25.88 (6.96)
	13 or more years	43	6.19 (1.88)	7.86 (5.04)	21.73 (7.24)	28.07 (6.3)
**Municipality Classification**	Predominantly Rural	91	4.02 (2.6)	13.14 (8.37)	10.44 (8.52)	23.17 (7.24)
	Mixed	19	5.89 (1.76)	9.0 (7.21)	14.21 (9.0)	26.37 (7.97)
	Predominantly Urban	78	5.57 (2.27)	8.99 (6.88)	19.43 (7.89)	27.18 (7.1)

The EFA reduction was suitable to use, since the Kaiser-Meyer-Olkin (KMO) statistic was meritorious (KMO = 0.81) and the Bartlett’s Test of Sphericity was significant (${\chi }^{2}$= 558.28, df 28, *p* < 0.001). The communalities ranged from 0.40 to 0.73, indicating that the variables had moderate to high correlations.

When extracting the factors, it was observed in the unrotated and the rotated solutions, that only two principal components remained in the test, explaining 60.99% of the variance. The first factor is composed of questions 1 to 4. It includes variables of self-efficacy in preventing tooth loss, selecting non-cariogenic food, and visiting the dentist in case of dental emergencies, calling this first factor “Tooth loss prevention” (TLP); its reliability was good (Cronbach’s alpha = 0.81). The second factor is composed of questions 5 to 8, including variables of self-efficacy in oral hygiene and precautions for drug use, calling this second factor “Self-care” (SC). For this set, Cronbach’s alpha coefficient was 0.74, indicating acceptable internal consistency. The correlation between the components was 0.53, suggesting a moderate association between them.

**Fig. 2:**
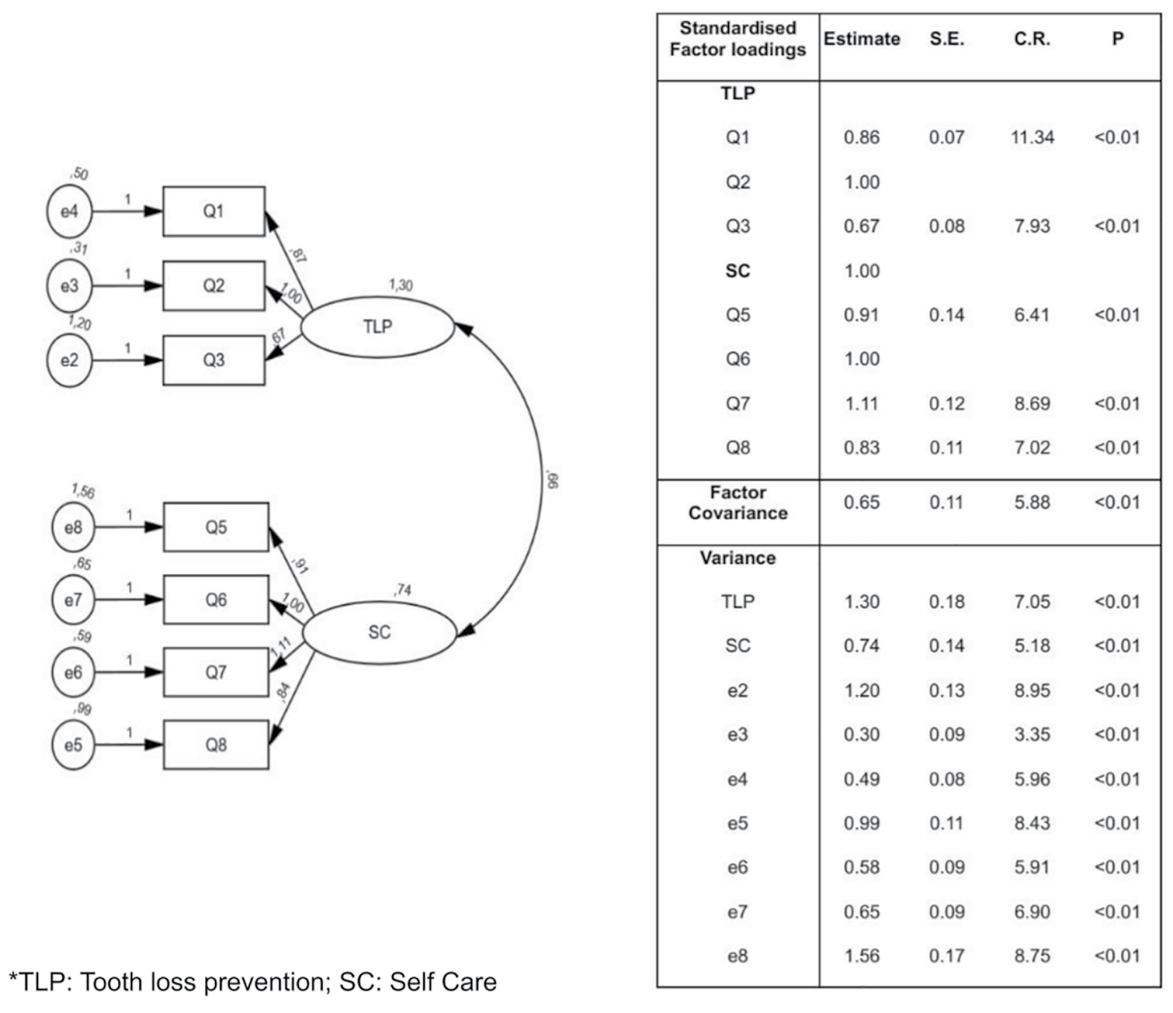
Path diagram and Parameter estimates of the Confirmatory Factor Analysis of the Oral Health Self-Efficacy Scale

Following the hypothetical model of two factors obtained from EFA, we continued with its confirmation through a CFA. The initial model had a lack of fit, with ${\chi }^{2}$= 3.35, CFI = 0.92, TLI = 0.88, and RMSEA = 0.11. Following the inspection of modification indices suggested by the software, we correlated a measurement error of Q3 and Q4 supported by the consideration that both questions evaluate the self-efficacy of going to the dentist in case of a dental emergency; with this modification, we obtained a good fit between the model and the observed data (${\chi }^{2}$= 1.74; CFI = 0.97; TLI = 0.96 and RMSEA = 0.06) [45]. The aforementioned modification assumes that whereas indicators Q3 and Q4 are related in part because of the shared influence of the factor Tooth Loss Prevention, some of their covariations are due to sources other than the common factor [45]. Considering that both indices evaluated similar aspects of the construct, the feasibility of eliminating one of these questions was evaluated through a principal component analysis to compare factor structures, which indicated that to maintain the 2-factor structure, question 4 should be removed. This decision was supported by the final CFA of the OHSES (Fig. 2), obtaining an excellent model fit (${\chi }^{2}$= 1.22, CFI 0.99, TLI = 0.99, RMSEA = 0.03), confirming the positive and not overlapping relation between the two constructs. In addition, as shown in Fig. 2, all the parameter estimates (factor loadings, factor covariance, and error variances) were statistically significant (*p* < 0.01). This outcome is consistent with the conclusion for the absence of localised areas of ill fit in the solution.

A reliability analysis showed the 7-question model (OHSES-7Sp) (Cronbach’s alpha = 0.82) to be as reliable as the 8-item OHSES (Cronbach alpha = 0.84). Test-retest reliability was calculated; the mean obtained in the first application of the questionnaire was 2.28 (SD 1.57) points, a value that increased during the second evaluation with a mean of 3.16 (SD 1.19). The single measures ICC was 0.411, which according to Fleiss (1986) [42], this coefficient indicates good agreement among the measures, suggesting consistency in the responses across different measures.

**Fig. 3:**
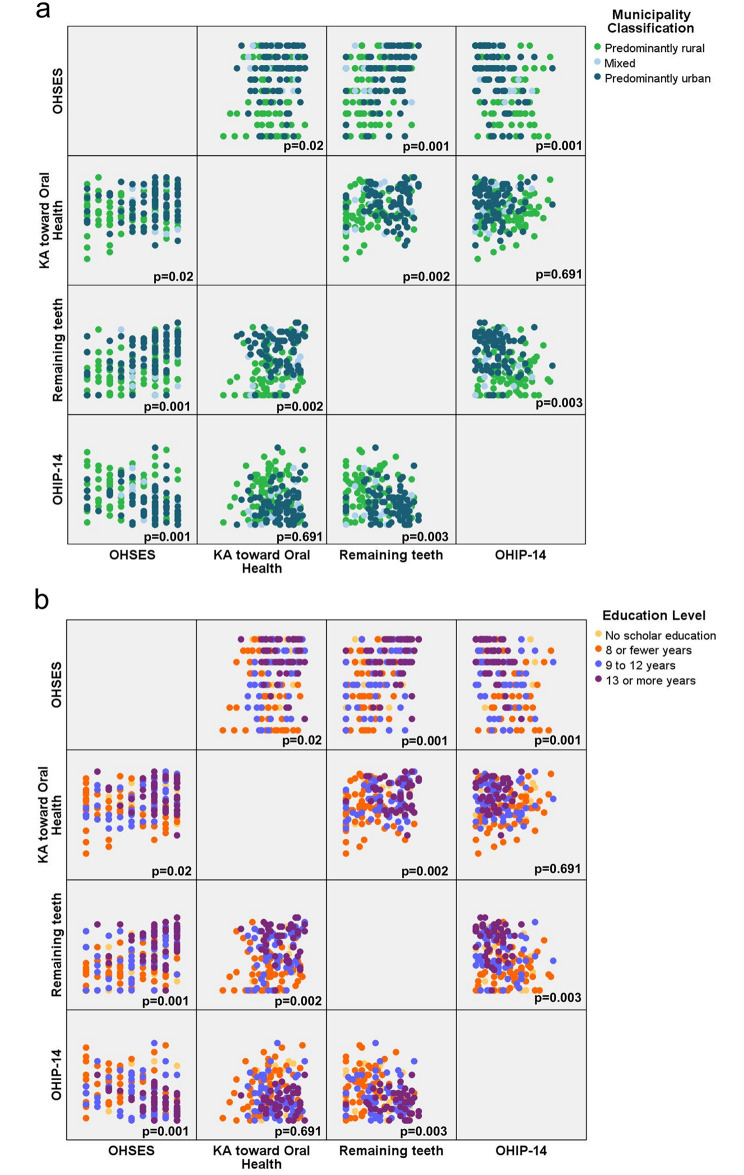
Scatter plot diagram for number of remaining teeth, knowledge and attitudes toward oral health, OHIP-14, and OHSES **(a)** Correlation of OHSES, knowledge and attitudes toward oral health, number of remaining teeth and OHIP-14 by municipality classification. **(b)** Correlation of OHSES, knowledge and attitudes toward oral health, number of remaining teeth and OHIP-14 by education level

When assessing the correlation between OHSES and sociodemographic characteristics, the OHSES exhibited a low positive correlation with educational level (*r* = 0.27, *p* < 0.01) and the type of municipality (*r* = 0.22, *p* < 0.01). Furthermore, it is worth noting that the latter two variables were found to be moderately correlated with each other (*r* = 0.56, *p* < 0.01). As hypothesised, there was a positive correlation between the variables: number of remaining teeth, Knowledge and attitudes toward oral health and OHSES; being moderately (*r* = 0.45, *p* < 0.01) and low positively correlated (*r* = 0.26, *p* < 0.001), respectively. The Oral Health Impact Profile − 14 Spanish had a moderately negative correlation (*r*= -0.36, *p* < 0.01) with the Spanish version of the Oral Health Self-efficacy Scale. The scatter plots illustrating the correlation between the number of remaining teeth, Knowledge and attitudes toward oral health, OHIP-14, and OHSES are presented in Fig. 3, categorized by type of municipality and educational level.

The final version of the OHSES 7-SP can be accessed as a related file (Related File 1).

## Discussion

The validation criteria for our study were deemed satisfactory, providing a robust foundation for the interpretation of our findings. Rigorous methodologies in the translation, adaptation, and validation of the OHSES, ensured the reliability and accuracy of the collected data, offering a reliable and valid measure of the oral health self-efficacy of Chilean older adults.

Given that multiples approaches can be employed to validate a scale, as constructs are not inherent to a scale but rather to its specific application in a particular sample [46], this study utilized three approaches to ensure that OHSES accurately and meaningfully measures oral health self-efficacy. Firstly, we addressed content validity through an expert panel, emphasizing the importance of a clear understanding of the construct. Experts in questionnaire validation, self-efficacy, or those involved in the care and management of older adults were selected. Additionally, for this judgment stage, a structured assessment form with expert feedback proved essential, with agreement indices considered alongside other factors to inform item rejection or modification [40]. The consideration of I-CVI, face validity, and expert comments contributed to a thorough evaluation of each item’s strengths and weaknesses. Experts distinguished between content relevance and wording clarity, leading to targeted improvements. The pilot testing phase included participants with diverse background, considering variations in age, educational levels, and place of residence. This approach aimed to achieve a broader representation, especially considering that in Chile younger older adults (i.e., 60–70 years) in urban areas tend to have a higher average educational level [47], this strategy, allowed for a more comprehensive evaluation of OHSES clarity and resulted in more precise item phrasing.

Secondly, data collection to assess the measurement model in our population of interest was conducted with EFA and CFA. The OHSES-7Sp demonstrated a simple structure with no correlated residuals. CFA and EFA addressed controversial items, such as question number 4, which was ultimately removed after demonstrating redundancy with question 3. The findings revealed that the two-factor model of the OHSES remained; however, the specific domains within each factor varied. These observed changes signify a comprehensive approach to assessing self-efficacy in oral health behaviours. Including the self-care domain acknowledges the importance of individual behaviours such as oral hygiene practices and medication use. Additionally, incorporating the prevention of tooth loss domain recognises the broader aspects related to diet, preventive measures, and prompt dental care, emphasising the significance of self-efficacy in retaining natural dentition.

Finally, the assessment of convergent and discriminant validity was based on theoretically related constructs previously mentioned in the literature. As hypothesized, our findings revealed a positive correlation between OHSES and key oral health indicators- number of remaining teeth, and knowledge and attitudes toward oral health- while inversely correlating with OHIP-14. These results provide convergent and divergent validity, aligning with existing research suggesting that self-efficacy functions as an intermediary variable between oral knowledge, attitude, and quality of life related to oral health [8–10, 18, 36]. This association implies that enhancements in knowledge may foster a positive attitude, augmenting oral health self-efficacy, prompting healthy behaviours, and ultimately, significantly contributing to an improved quality of life related to oral health. However, further exploration of these factors in future research is needed for a comprehensive understanding of this intricate interplay.

Turning our attention to sociodemographic factors, previous research has identified significant sociodemographic (i.e., sex and level of education) and dental status differences in oral health attitudes and OHSES [8]. However, to the best of our knowledge, the impact of the place of residence on oral health self-efficacy remains unexplored. Health disparities between rural and urban populations are a global concern, leading to variations in morbidity rates, access to oral healthcare, prevalence of missing teeth, and self-perceived oral health [48, 49]. This difference can be attributed to differences in oral health concepts, higher social acceptance of edentulism, and poorer health behaviours and lifestyle choices in rural settings [49]. To enhance the representativeness of our study, we deliberately included participants from diverse types of municipalities, encompassing urban, mixed (combining rural and urban attributes), and rural areas. This approach enabled us to capture a broad spectrum of response patterns and reaffirmed the impact of socio-demographic characteristics on oral health among older adults in these diverse settings. Specifically, individuals living in rural areas exhibited poorer outcomes across the studied variables (education level, number of remaining teeth, knowledge and attitudes toward oral health, OHIP-14 and OHSES). The results revealed that most participants reported moderate levels of self-efficacy regarding oral health behaviours (4.85 ± 0.38). However, these levels decreased in those with lower levels of education (3.83 ± 2.6) and those living predominantly in rural areas (4.02 ± 2.6). These findings are consistent with the results obtained in previous studies applying OHSES in rural and urban communities of older adults, revealing slightly lower levels in the rural population (83.1% vs. 84.37%, respectively) [8, 18]. This decline in perception of self-efficacy, indicates a lack of confidence and belief in their ability to manage their own oral health and a decrease in their oral health involvement in decision-making about their healthcare [16].

The oral health status is impacted by diverse factors, encompassing social, physical, functional, and emotional aspects [9]. Despite advances in prevention and treatment [1, 19], the issue of tooth loss and edentulousness continues to be a concern among older adults, having complete edentulism a global prevalence in older adults estimated at 23%, slightly surpassing the 17.1% in Chile [2, 30], posing a societal challenge and emphasising the need for ongoing efforts to promote oral health for healthy ageing. Factors such as patients’ dietary habits, self-care, and using preventive services for preventing tooth loss and the maintenance of oral health have been addressed [50, 51]. Importantly, these factors are inherently embedded within the constructs identified in OHSES. Consequently, OHSES emerges as a valuable tool for evaluating patients’ levels in these crucial aspects, offering potential new insights and strategies for enhancing oral health status.

A limitation of our study was the test-retest reliability: although the stability of the responses in our sample was acceptable [42], the result was lower than expected. A possible explanation for this might be that questionnaires that measure more subjective constructs are less reliable [52], taking into account that self-efficacy it´s a multifactorial construct and that people’s beliefs about their abilities vary between domains and situations, rather than being uniform between tasks and contexts as a general trait [53]. Therefore, is possible that exposure to the first test made them reflect on their abilities, increasing their self-efficacy in the retest. Furthermore, this enhancement could be linked to artefactual factors like the Hawthorne effect. In addition, the first test included the assessment of the knowledge and attitudes towards oral health and OHIP-14 Sp; increasing the application time could decrease the attention in the first attempt [54]. These participants may also have a lower household income and education than the English validation sample, which is consistent with Tourangeau´s findings [52], where the respondent´s adverse sociodemographic conditions reduce the test-retest reliability. One additional limitation associated with the study´s design is the inability to establish causal relationships of the construct and other factors related to behaviour change, therefore, conducting longitudinal studies is necessary. The decision to implement the OHSES as an interviewer-administered tool allowed us to prevent missing data and eased the administration to individuals with diverse educational backgrounds, including those who are illiterate. This approach enabled us to assess the comprehensibility of the scale, its construct, and its applicability in the Chilean older adult population. One potential bias in this type of administration is the tendency to social-desirability bias. However, previous studies have provided evidence suggesting that administration formats do not have a meaningful effect on measurements of patient-reported outcomes [55].

Nonetheless, although our data may possess certain limitations, we firmly believe that the outcomes of this study significantly enhance oral health research across multiple dimensions. Using convenience sampling from regional and municipal databases made it possible to incorporate a comprehensive and representative sample from the area. This sample comes from diverse demographic backgrounds, including variations in terms of sex, age, ethnicity, educational attainment, and residential location. This inclusiveness ensures the representation of a diverse population. Additionally, we have observed considerable response variations, facilitating an assessment of the scale’s structure and precision, thus effectively fulfilling the study’s objectives. Furthermore, the sample size surpasses the theoretically established minimum requirements and meets the criteria for sampling adequacy necessary for the EFA and CFA performed in this study. Moreover, adopting multiple indices for model evaluation (including assessment of absolute fit, adjustment for model parsimony, and relative fit compared to a null model) has provided a more conservative and reliable assessment of the OHSES-7Sp solution.

In summary, the validation of OHSES in Chilean older adults, provide a valuable new tool that provides crucial insights into patients’ convictions and self-assurance in managing their oral health. The integration of this patient-reported outcome enhances our understanding of the various factors contributing to oral diseases among older adults, placing oral health within the broader framework of personal and societal well-being. The absence of routine and preventive oral health care within the current healthcare system in Chile [56] underscores the urgency for initiatives tailored to address the unique needs of older adults. The potential application of OHSES in this demographic, could emerge as a potent instrument for identifying areas of low self-efficacy, facilitating the design of tailored interventions to enhance oral health outcomes, this approach could optimize resources by focusing on areas of greater need, guiding the development of preventive programs, and contributing to ameliorating the oral health challenges faced by the ageing population in Chile. Moreover, the OHSES holds the potential to serve as a significant endpoint in dental research studies, thus supporting evidence-based decision-making and the evaluation of intervention effectiveness [19, 20]. Its validation further enables both national and international comparisons. We suggest implementing longitudinal studies to increase the degree of evidence and assess the relationship between the OHSES and clinical variables.

## Conclusion

The findings of this study confirm the psychometric properties (i.e., validity and reliability) of the Oral Health Self-Efficacy Scale (OHSES) when applied to older adults in Chile. Furthermore, this is the first study that explored the validity of the OHSES. The utilisation of the OHSES-Sp in dental interventions is expected to serve as a valuable tool for assessing self-efficacy levels in this population. Additionally, the implementation of the OHSES-Sp enables the collection of data for use in national and international comparisons, enhancing the understanding and evaluation of self-efficacy in oral health across different contexts.

## Data Availability

The datasets used and/or analysed during the current study are available from the corresponding author upon reasonable request.

## Abbreviations

OHSES: Oral Health Self-Efficacy Scale
OHSES (Sp): Spanish version of Oral Health Self-Efficacy Scale
OHIP-14: Oral Health Impact Profile
TEGO: Geriatric Dental Specialties Tele platform
DSES: Dental Self-Efficacy Scale
SESS: Self-efficacy scale for self-care
OHSE: Oral Hygiene-related Self-Efficacy
GSEOH: Geriatric Self-Efficacy Scale for Oral Health
SENAMA: National Senior Citizen Service
I-CVI: Item content validity index
ICC: Intraclass correlation coefficient
EFA: Exploratory factor analysis
KMO: Kaiser-Meyer-Olkin statistic
PCA: Principal component analysis
CFA: Confirmatory factor analysis
ML: Maximum likelihood
CFI: Comparative fit index
TLI: Tucker-Lewis index
RMSEA: The root means the square error of approximation
SPSS: Statistical Software for the Social Science
TLP: Tooth loss prevention
SC: Self-care
